# Research on the Mechanism of Micro-Water Jet-Guided Laser Precision Drilling in Metal Sheet

**DOI:** 10.3390/mi12030343

**Published:** 2021-03-23

**Authors:** Yinuo Zhang, Hongchao Qiao, Jibin Zhao, Zhihe Cao

**Affiliations:** 1 State Key Laboratory of Robotics, Shenyang Institute of Automation, Chinese Academy of Sciences, Shenyang 110016, China; zhangyinuo@sia.cn (Y.Z.); caozhihe@sia.cn (Z.C.); 2 Institutes for Robotics and Intelligent Manufacturing, Chinese Academy of Sciences, Shenyang 110169, China; 3 University of Chinese Academy of Sciences, Beijing 100049, China

**Keywords:** water jet-guided laser processing, microporous manufacturing, finite element simulation, taper, metal material

## Abstract

As the microporous structure has been widely used in the field of precision machining, at the same time, the requirements for the quality of microporous machining are continuously increasing. Water jet-guide laser processing technology (WJGL) has been gradually applied for its high machining precision. However, there are a few researches on the heat conduction process of WJGL processing metal materials. Therefore, it is of great significance to study the transient thermal effect of metal materials and the mechanism of material removal to improve the processing quality. In order to explore the heat conduction model of WJGL processing metal materials, this paper is based on the “element birth and death” technique in the finite element method, and the three-dimensional transient temperature field of four typical metal materials (titanium alloy, stainless steel, aluminum alloy, copper) and material removal model are established. Under this model, the removal mechanism of different metal materials and the influence of different process parameters on the temperature field distribution of the material are studied, and the influence of fixed-position drilling and helix drilling on the microporous morphology is compared. The results show that copper and aluminum alloys can obtain a larger depth-to-diameter ratio and a smaller hole taper. Titanium alloy and stainless steel have better hole roundness, lower hole edge temperature, and smaller thermal deformation. Hole roundness error and hole taper decrease with the increase of laser power. The roundness error of each material is reduced to within 10 μm when the laser power is 10 W, and the average hole taper is 8.73°.

## 1. Introduction

With the advancement of science and technology, microporous structures have broad applications in aerospace, high-sensitivity optical fiber sensors, consumer electronics, three-dimensional integrated circuit packaging and other fields. At the same time, the requirements for microporous processing quality have been constantly improved [1]. However, traditional microporous processing methods have certain limitations: the depth-diameter ratio of microporous formed by mechanical drilling is very small, and is not suitable for processing brittle and hard materials [2]. EDM drilling has low efficiency and can only process conductive material, and the loss of electrodes makes the operation cost of this technology high [3,4,5]. The applicable conditions for electron beam and focused ion beam processing of micro holes are relatively harsh and the needed equipment is expensive [6]. It is difficult to control the corrosion direction when machining micro holes by electrochemical methods, resulting in a relatively smaller diameter of the processed micro holes [7]. In the process of electro hydraulic beam micro hole machining, chip removal is difficult and there are some problems such as easy contamination of the processed sample with the electrolyte [8]. At present, laser processing technology has been widely applied to the microporous processing of metal materials, but for non-metallic materials, especially hard, brittle, and transparent materials, there is no large amount of free electrons, so its absorption rate for continuous laser and long pulse laser is low. It is difficult to achieve high-efficiency processing and the processed microporous have obvious thermal effects, recast layers, and other defects [9,10]. In recent years, ultrafast laser processing technology has gradually entered the field of microporous manufacturing, but it is difficult for this technology to take into account the processing accuracy, quality, and efficiency at the same time, which restricts its further development and application.

Water jet-guided laser processing technology is a composite processing technology, and this modern processing method has been increasingly valued by the precision processing industry [11,12]. This technology combines the nanosecond laser technology with the water jet cooling technology, and makes use of the difference of optical characteristics when light propagates in water and air, so as to achieve the guidance and broken line transmission of laser beam in the water jet. During the processing, the water jet is used as an optical fiber to transmit the laser to the surface of the work piece, and remove excess heat and residue on the surface of the work piece during processing, so as to clean and tidy the kerf, and solve some disadvantages of traditional laser processing [13,14]. High-speed water jet plays an important role in this technology. As the light source is a high repetition frequency pulsed laser, the temperature at the laser irradiation point can be raised rapidly to the decomposition temperature, and the processing point can be cooled rapidly by water jet after the pulse ends, so as to reduce the thermal damage caused by the accumulation of irradiated heat of the beam at the same processing point. Compared with the traditional laser processing technology, the water jet-guided laser processing technology has significant advantages and is gradually applied in the high-precision microporous processing.

In recent years, this technology has attracted more and more attention and research from researchers. Chen et al. [15] used the finite difference method to simulate the space-time distribution of the optical power density and plasma density in the axial direction of the ultrashort pulse during transmission. The dynamic process of ultra-short pulse transmission in water is analyzed, and the research shows that too high laser power density will make the water jet reach the breakdown threshold and generate plasma, which defocus the laser pulse, leading to defects in processing. Lu et al. [16] studied the laser energy distribution in the water fiber during processing and found that the energy distribution of the laser was improved due to the influence of the water jet, the effective laser processing threshold became larger, and the energy distribution became nearly uniform. Sun et al. [17] compared the surface morphologies of water jet-guided laser processing and water-assisted laser processing of monocrystalline silicon wafers, and found that the channels processed by water-guided lasers were wide and shallow, with a “V” shape. The surface of the channel was clean, less slag, no burr, and small heat-affected zone, applied for the special problem of scribing round cutting processing. In summary, water jet-guided laser processing has great potential in the field of precision processing.

At present, there are few researches on the mechanism of using water jet-guided laser to process metal materials. Metals and alloys cover almost all materials used in special processing fields, especially aluminum alloys, titanium alloys, copper, and stainless steel. Therefore, it is of great significance to study the transient thermal effect of water jet-guided laser processing of metal materials and the mechanism of material removal to improve the processing quality and reduce the range of process parameters.

Based on the “element birth and death” technique in the finite element method, the three-dimensional transient temperature field and subsequent material removal model of multi-materials are established. Under this model, the above four materials are divided into two categories: high hardness, low thermal conductivity (titanium alloy and stainless steel) and low hardness, high thermal conductivity (aluminum alloy and copper). The morphology characteristics of microporous processed by different metal materials were studied, and the influence of fixed-position drilling and spiral drilling on the morphology of the holes was compared. The results show that water jet-guided laser processing of microporous can obtain a larger depth-to-diameter ratio while ensuring better processing accuracy. Adjusting the laser power can reduce the taper of the microporous, and the effect of water jet on the material cooling by strong convection is remarkable, which plays an important role in ensuring the roundness of the hole.

## 2. Experimental Setup

### 2.1. Material Properties

Four different metal sheet materials were used in the experiment, 6061 aluminum alloy (AlMg1SiCu), TC4 titanium alloy (Ti6Al4V), pure copper (T2), 304 stainless steel (06Cr19Ni10), respectively. The main chemical composition of them is shown in Table 1. The sample size is 50 mm × 50 mm × 0.2 mm. All the samples were cleaned with isopropyl alcohol before processing. Table 2 shows the thermodynamic performance parameters of each material.

### 2.2. Experimental Process

The laser source used in the experiments is Nd:YAG solid-state pulsed laser with a wavelength of 532 nm. Its maximum average power is 30 W and the output beam has a Gaussian distribution. During the experiment, the output frequency was 40 kHz, and the pulse width was 0.3 µs. We developed an experimental platform of WJGL, including the water circulation system, the water-laser coupling unit, and the three-dimensional working platform. In order to reduce the absorption of laser energy by water, the experimental water was filtered, deionized, and degassed.

**Figure 1:**
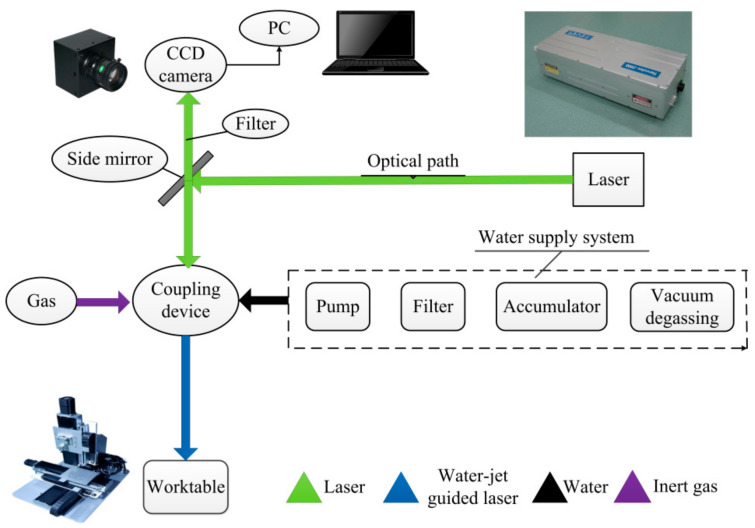
The schematic diagram of water jet-guide laser machining experimental platform.

**Figure 2:**
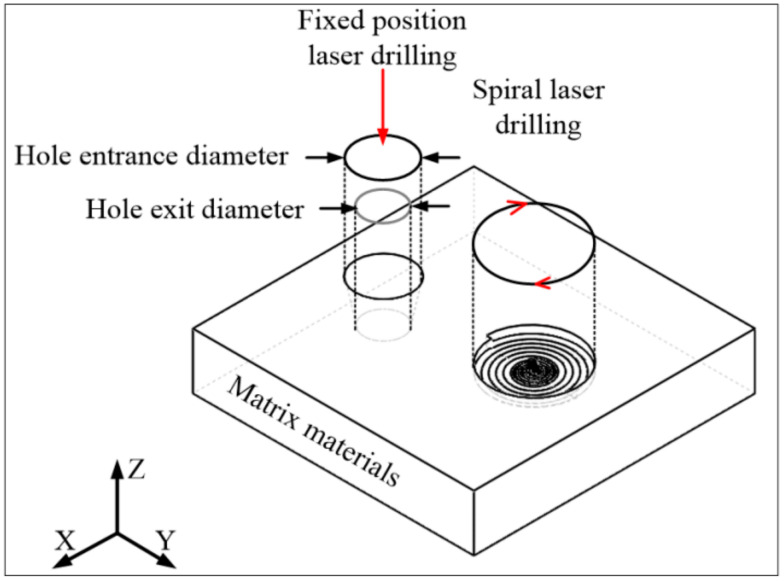
Strategies used for water jet-guided laser machining.

In the experiment, the work piece was fixed on the X-Y plane of the three-dimensional platform, and the water-laser coupling unit was installed on the Z axis. The laser beam was focused into a water nozzle with a diameter of 70 µm and coupled with the water jet. The laser energy was guided by the water jet to the work piece to complete the material processing. The schematic diagram of each component of the experimental platform is shown in Figure 1. Figure 2 shows the two processing schemes of the drilling experiment, including fixed-position drilling and helical drilling.

After drilling by the water jet-guided laser processing, the samples were dried by clean compressed air. The surface morphology was studied to understand the machining accuracy. The 3D surface morphology was measured by the Bruker Contour GT-K optical microscope (Bruker, Madison, WI, USA) with a 50× objective, scanning electron microscope (Zeiss, Oberkochen, Germany), and model ZEISS EVO MA 10/LS 10. Hole diameter data were directly obtained from the measured 3D data. For all the data measurements, each micro-hole was measured 6 times, and the test results were averaged.

## 3. Finite Element Analysis

### 3.1. Geometric Model

**Figure 3:**
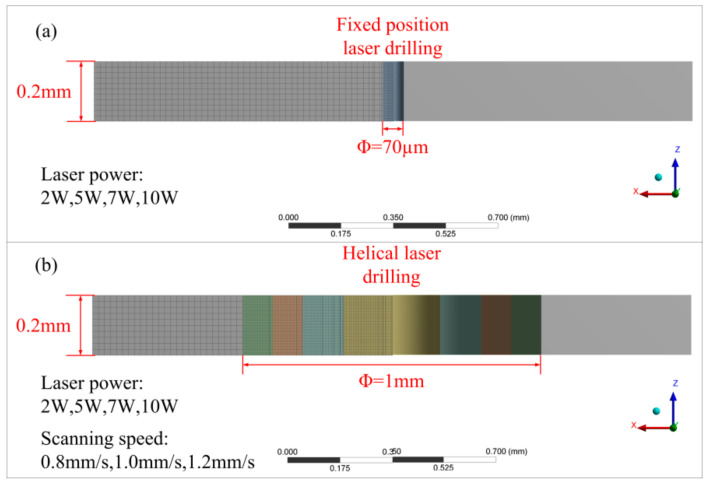
Materials geometric model used for simulation: (**a**) fixed-position drilling and (**b**) helical drilling.

The simulation is aimed at studying the processing mechanism and the heat transfer process of metal materials processed by WJGL. The model size is designed as 2 mm × 2 mm × 0.2 mm, which is much larger than the size of the processed hole, and it can make the best of the heat transfer process analysis of the material. Due to the ablative depth is insufficient, the main function of water jet is heat transfer to the machined surface. This article considers the cooling effect of jets as a strong convective cooling coefficient and ignores the water flow. The laser power is applied to the model surface, and the laser heat source diameter is 70 µm. The laser energy transmission direction is parallel to the Z axis. Table 3 shows the setting parameters during simulation. Figure 3 shows the simulation model and specific scheme.

In order to simulate the transient thermal response and subsequent material removal, the “element birth and death” in ANSYS finite element analysis software was adopted. Therefore, the material removal conditions have a great impact on the accuracy of the simulation. Assumptions about material removal in the model include:

- As the temperature rises, the material will melt and pyrolysis when it reaches the melting point. Considering the impact of the water jet, the molten material should be washed away after reaching the melt temperature [18]. Since the material is always covered by water jet during the processing and the deionized ultrapure water is used in this experiment, the removal environment of materials is always oxygen-free, so there is no phase transition caused by material oxidation.
- The sheet material is homogeneous and isotropic in nature. The laser pulse width is much smaller than the entire pulse cycle, and the evaporated material is transparent and does not interfere with the incoming laser beam. Therefore the metal vapor is optically thin so that its absorption of the high-energy beam is negligible.
- After every time step is calculated, the temperature of each unit on the workpiece is detected. Once the temperature of the unit reaches material removal temperature, it is deleted. The unit will no longer participate in the calculation at the beginning of the next time step. At the same time, a new boundary is formed, and the boundary conditions are reset. At this time, the heat flow will be applied to the surface of the living element.

### 3.2. Governing Equations

In this study, transient heat conduction is the main consideration. Therefore, the governing equation is time-dependent heat conduction equation [19]:

(1) $$\rho C\frac{\partial T}{\partial t}=\nabla \cdot \left(\lambda \nabla T\right)$$

where ρ, C, and λ are density (kg/m^3^), specific heat (J/(kg·K)), and thermal conductivity (W/(m·K)).

At the top surface, where the laser flux and water-cooling effect are applied, the convective boundary conditions are considered in equation [19]:

(2) $$-\lambda \frac{\partial T}{\partial n}=q-h_{1}\left(T-T_{\infty }\right)$$

As the whole of the model is completely covered by water jet, other surfaces except the top were considered as natural convection boundary condition of water, as shown in equation [19]:

(3) $$\lambda \frac{\partial T}{\partial n}=h_{2}\left(T-T_{\infty }\right)$$

In the above equations, n is the normal vector of surface, $h_{1}$ and $h_{2}$, T and $T_{\infty }$ denote the water severe convection heat transfer coefficient, water natural convection heat transfer coefficient, the cell temperature and the ambient temperature, respectively. q is the laser absorption heat flux (W/mm^2^).

The boundary conditions can be given by Equations (4)–(6):

(4) $$z=0,F_{0}=\frac{P\left(1-R\right)}{A}=-\lambda \frac{\partial T}{\partial z}$$

(5) $$z=\infty ,\lambda \frac{\partial T}{\partial z}=0$$

(6) $$t=0,T=T_{\infty }$$

where λ is the thermal conductivity (W/(m·K)), z is the depth of laser action (m), $F_{0}$ is the peak power density (W/m^2^), P peak power (W), R is the reflectivity of the material surface, A is the spot area (m^2^), $T_{\infty }$ is the initial temperature (K).

The laser emitted by the laser source has a Gaussian distribution. However, the laser beam guided by the water jet as a multi-mode fiber can change the laser distribution into the uniform distribution of all sections along the direction of the water jet [20]. Therefore, the laser absorption heat flux is considered to be of uniform distribution:

(7) $$q\left(t\right)=\frac{P\left(t\right)}{\left(\frac{\pi }{4}D^{2}\right)}$$

where P is the peak power of pulsed laser (W) and D is the diameter of water jet (m), t is the processing time (s).

**Figure 4:**
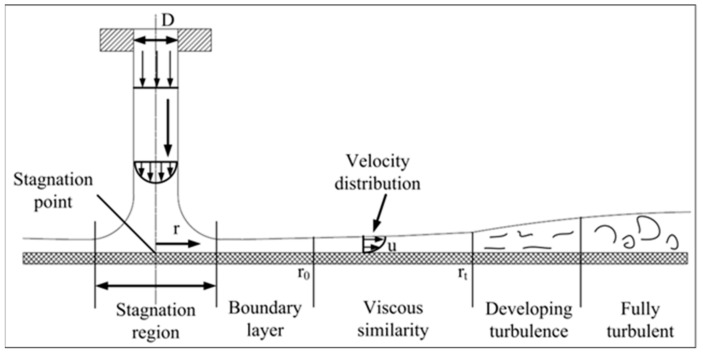
Schematic diagram of jet flow field of circular nozzle [21].

The strong convective cooling effect of water jet is the key of water jet laser processing. The laser action area and the contact surface between the material and the laser are all subjected to the cooling and convection action of the high-pressure water jet. As shown in Figure 4, when the jet reaches the wall of the impacted object, the fluid spreads around the wall form a wall-mounted jet area. The area where the impacted wall faces the nozzle becomes the stagnation zone, and the point corresponding to the center of the jet is called stagnation point where the local heat transfer intensity is the highest. With the increase of r, the local heat transfer coefficient decreases monotonously from the highest value at the stagnation point to the surrounding area, and finally the downward trend gradually slows down. The calculation formula for the range of the laminar heat exchange zone formed by the water jet contacting the material is as follows [21]:

(8) $$r_{0}=0.1773DRe^{\frac{1}{3}}$$

The Nμ empirical formula is [21]:

(9) $$N\mu =0.632Re^{\frac{1}{2}}Pr^{\frac{1}{3}}{\left(\frac{D}{r}\right)}^{\frac{1}{2}}$$

where $Re=\frac{V\cdot l}{\upsilon }$, $Pr=\frac{c_{w}\cdot \eta }{\lambda }$, $V=\varphi {\left(\frac{2P_{w}}{\rho_{w}}\right)}^{\frac{1}{2}}$.

where D is the diameter of water jet at the nozzle (m). Re and Pr are Reynold number and Prandtl number, respectively. r is the distance between heat transfer zone and stagnation point (m). $V,\upsilon ,\eta ,c_{w},\rho_{w},P_{w}$ and φ are flow velocity (m/s), the motion viscosity coefficient of water (m^2^/s), kinetic viscosity coefficient of water (Pa·s), the specific heat capacity of water (J/kg °C), the density of water (kg/m^3^), the water pressure (MPa), and the pressure loss factor, respectively.  Nμ is Nusselt number.

The relevant parameter values for calculating the heat transfer coefficient of water flow are shown in Table 4. The calculated heat transfer radius of the laminar flow zone $r_{0}$ is 614 µm, the position Nu is 142. From the formula $Nu=\frac{hr}{\lambda }$, the local heat transfer coefficient h leaving the stagnation point $r_{0}$ is 0.14 (W/(mm^2^·K)).

In the region where the viscosity of fluid increases gradually, the heat transfer capacity of water jet and material surface also changes accordingly. The Nu calculation formula is [22]:

(10) $$Nu=\frac{0.407{Re}^{\frac{1}{3}}{Pr}^{\frac{1}{3}}{\left(\frac{D}{r}\right)}^{\frac{2}{3}}}{{\left[0.1713{\left(\frac{D}{r}\right)}^{2}+\frac{5.147r}{Re\;D}\right]}^{\frac{2}{3}}{\left[\frac{1}{2}{\left(\frac{r}{D}\right)}^{2}+C_{3}\right]}^{\frac{1}{3}}}$$

(11) $$C_{3}=\frac{0.267{\left(\frac{D}{r_{0}}\right)}^{\frac{1}{2}}}{{\left[0.1713{\left(\frac{D}{r_{0}}\right)}^{2}+\frac{5.147r_{0}}{Re\;D}\right]}^{2}}-\frac{1}{2}\left(\frac{r_{0}}{D}\right)$$

After the water jet contacts the material to exchange heat sufficiently, a turbulent heat exchange zone is finally formed. $r_{t}$ is the distance between turbulence zone and stagnation point (m). The calculation formula for the range of this zone is as follows [22]:

(12) $$\left(\frac{r_{t}}{D}\right){Re}^{0.422}=1.2\times 10^{3}$$

The Nu empirical formula is [22]:

(13) $$Nu=0.195{Re}^{0.98}{Pr}^{0.38}{\left(\frac{D}{r}\right)}^{1.95}$$

## 4. Results and Discussion

### 4.1. Simulation Results

**Figure 5:**
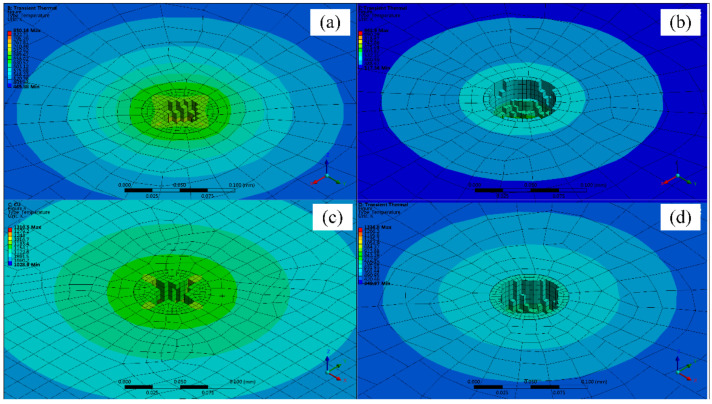
The drilling simulation of hole shape: (**a**) aluminum alloy, (**b**) titanium alloy, (**c**) copper, (**d**) stainless steel.

**Figure 6:**
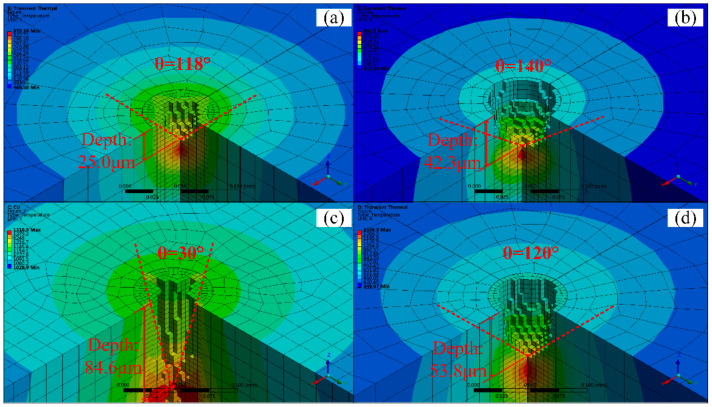
The drilling simulation of hole interior shape: (**a**) aluminum alloy, (**b**) titanium alloy, (**c**) copper, (**d**) stainless steel.

Figure 5 and Figure 6a–d are the simulation of fixed-position drilling of 6061 aluminum alloy (AlMg1SiCu), TC4 titanium alloy (Ti6Al4V), pure copper (T2), and 304 stainless steel (06Cr19Ni10) processed by WJGL, respectively. The laser power is 10 W, and the drilling time is 1.2 s. It can be seen from the figure that the processing depth of copper is the deepest in the same processing time, at 84.6 μm. The heat transfer is the fastest in the radial and axial directions. However, the residual heat around the hole after processing is higher, the temperature is about 1000 K, and the thermal deformation is serious. The machining depth and the diameter of aluminum alloy is 25 µm and 56 µm respectively, which is the smallest among the four materials. It shows that the thermal physical properties of the material directly affect the processing effect. Titanium alloy and stainless steel have a larger hole diameter, about 70 μm, the roundness of the hole shape is kept better, the hole edge temperature is lower, about room temperature, and the thermal deformation is small.

By the simulation results, it can be seen that the water jet-guided laser processing of copper, aluminum alloy, and other materials with low hardness and high thermal conductivity can obtain a larger depth-to-diameter ratio, and the hole taper is small, while for titanium alloy, stainless steel, and other materials with high hardness and low thermal conductivity. The water jet-guided laser drilling has a vertical hole wall shape, better roundness, lower temperature at the edge of the hole, and less thermal deformation. From the above research, it can be found that water jet-guided laser processing materials with high hardness and low thermal conductivity (e.g., TC4 titanium alloy, 304 stainless steel) have good hole-making ability, and can ensure better hole roundness while possessing higher processing efficiency, which is beneficial to forming a micro-hole structure with flat bottom and vertical side wall, and has higher precision processing ability.

**Figure 7:**
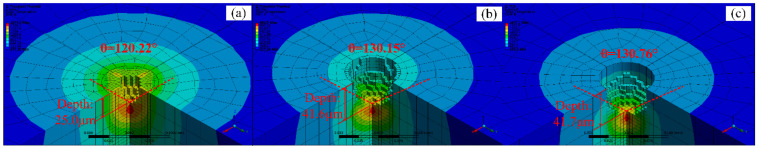
Simulation results of water jet-guided laser fixed-position drilling of titanium alloy: (**a**) the laser power is 5 W and the repetition frequency is 40 kHz, (**b**) the laser power is 9 W and the repetition frequency is 40 kHz, (**c**) the laser power is 9 W and the repetition frequency is 80 kHz.

Taking titanium alloy as an example, this article carries out a fixed-position drilling simulation of changing the laser power and changing the laser repetition frequency of the material, as shown in Figure 7, where the processing time is 1.2 s. Comparing Figure 7a,b, it can be seen that when drilling with low laser power, the hole diameter and hole depth are smaller, and the sharp angle at the bottom of the hole is as small as 120.22°. Comparing Figure 7b,c, it can be seen that the laser repetition frequency has little effect on the shape of the hole bottom. There is a slight increase in diameter at high repetition rates, but there is almost no change in depth and hole taper. The depth is changed from 41.6 μm to 41.7 μm, and the sharp corner at the bottom of the hole is changed from 130.15° to 130.76°. The temperature around the hole is lower after drilling, closing to room temperature. It is obvious that the strong convection heat transfer effect of the water jet is significant in the WJGL, which plays an important role in ensuring the shape error of the hole.

This is due to the high-intensity and high-energy density nanosecond pulsed laser in the water jet-guided laser processing produces superheated liquid. In a short time, a large number of bubbles in the liquid nucleate uniformly and increase exponentially. When the temperature is close to the critical temperature of the liquid into gas, the superheated liquid turned into gas in a large amount and exploded marked by droplet throwing. However, the phase change explosion of the material has the property of delay, which generally occurs after the end of the pulse. At this time, the molten material is washed and the surface of the material is cooled under the action of the water jet, so there is no heat accumulation during the processing, and the processing area is always room temperature.

**Figure 8:**
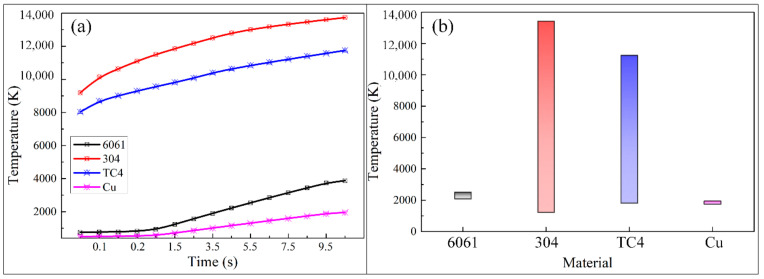
Simulation of the internal heat accumulation process of the material after the water jet-guided laser fixed-position drilling: (**a**) the change in temperature with processing time, (**b**) temperature difference at the end of processing (unset “element birth and death”).

Figure 8a simulates the heat accumulation process inside the material after the fixed-position drilling of the WJGL for 10 s. It can be seen from the figure that the temperature of titanium alloy and stainless steel rise rapidly in the initial stage of laser action, and the material removal temperature can be reached rapidly. As the processing time goes by, the temperature of the material shows an upward trend, while the temperature growth rate shows a downward trend, and the temperature increases slowly and gradually stabilizes at about 5 s. On the contrary, the temperature rise of aluminum alloy and copper is relatively slow at the initial stage of laser action, with the temperature growth rate almost 0, and it rises rapidly at about 1 s, after which the growth rate tends to be stable. Figure 8b is the temperature distribution on the surface of the model after the laser pulse stopped. The temperature difference on the surface of the aluminum alloy and copper is not large, and the minimum temperature and maximum temperature are about 2000 K, indicating that the heat transfer is faster and the whole model is at high temperature after processing. The surface temperature difference between stainless steel and titanium alloy materials is relatively large, the temperature difference on the surface of stainless steel is more than 12,000 K, and the minimum temperature is relatively low. It can be found that the strong cooling effect of the water jet in the WJGL will make a stable temperature difference on the surface of the material. In contrast, the material with lower thermal conductivity has the best cooling effect and the edge temperature of the material is lower.

**Figure 9:**
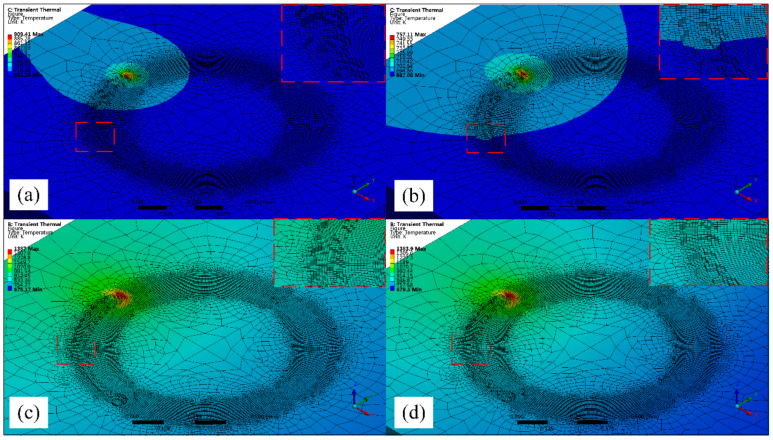
Helical drilling simulation of water jet-guide laser processing technology (WJGL):(**a**) scanning aluminum alloy at 0.8 mm/s, (**b**) scanning aluminum alloy at 1.2 mm/s, (**c**) scanning titanium alloy at 0.8 mm/s, and (**d**) scanning titanium alloy at 1.2 mm/s.

Figure 9 is the simulation study of a quarter spiral punch processed by WJGL. When aluminum alloy is processed at a single scan and low speed, there are obvious traces of discontinuous ablation pits at the bottom of the groove. While at high speed, the bottom of the groove is relatively flat and the processing quality is better. When the titanium alloy is processed by a single scan, the speed change has no obvious influence on the groove morphology. Discontinuous ablative pits appeared at the bottom of the groove at the early stage of high-speed processing. With the increase of time, the shape of the groove bottom was gradually flattened. By comparing the simulation research of the two materials, it can be found that the groove depth is deeper and the groove width is slightly wider after aluminum alloy processing. When scanning at low speed for both materials, the groove width is slightly larger than when scanning at high speed.

### 4.2. Experimental Results

**Figure 10:**
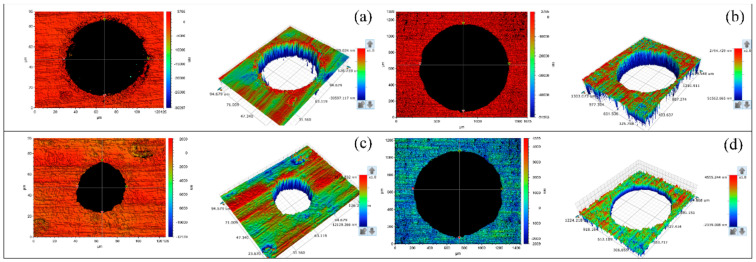
Surface morphology of 304 stainless steel after water jet-guided laser drilling in different paths at the same laser power: (**a**) fixed position drilling upper surface, (**b**) helical drilling upper surface, (**c**) fixed position drilling lower surface, (**d**) helical drilling lower surface.

**Figure 11:**
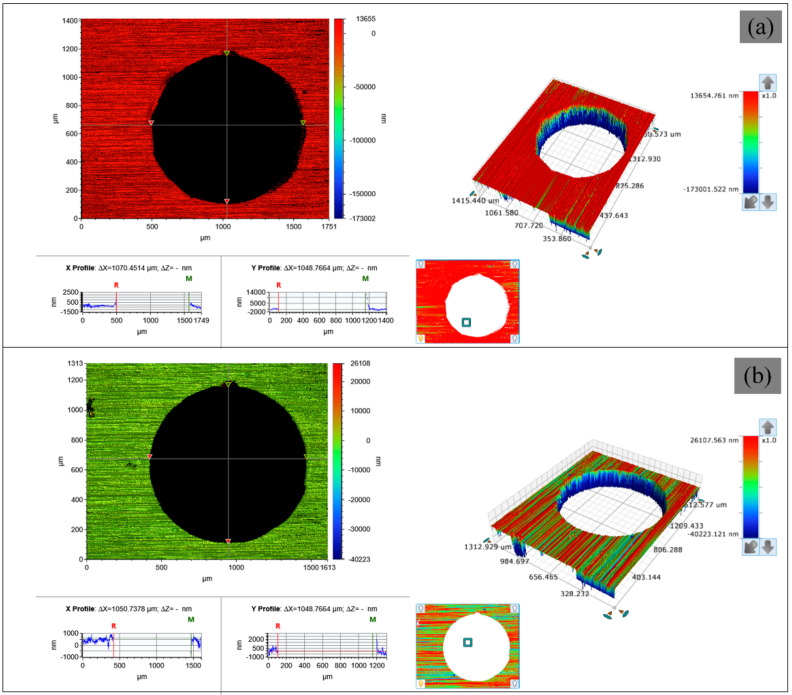
Surface morphology of 6061 aluminum alloy drilled by WJGL at different speeds under the same laser power in accordance with helical drilling method: (**a**) 0.8 mm/s, (**b**) 1.2 mm/s.

In order to further study the technological characteristics of water jet-guided laser processing of microporous, combined with the finite element simulation, the four kinds of metal materials in the different laser power, different scanning speed, and different drilling paths were studied. Figure 10 shows the surface morphology of 304 stainless steel after drilling holes in different paths by a WJGL at the same laser power. It can be seen from the figure that due to the small hole diameter of the fixed-position drilling hole, the material melt jet destroys the shape of the hole wall, and there is a small amount of melt accumulation around the laser entrance face, and the laser exit face shape is approximately ellipse. Helical drilling can obtain a good hole shape, and both the laser incident surface and the laser exit surface are round holes with high roundness. Figure 11 shows the surface morphology of 6061 aluminum alloy after being drilled by WJGL under the same laser power and different scanning speeds in a helical drilling path. The study found that the hole shape is relatively good when scanning at high speed. The scanning speed has a little effect on the aperture. The aperture is slightly larger when scanning at low speeds, so increasing the scanning speed during processing can obtain microporous with higher roundness.

**Figure 12:**
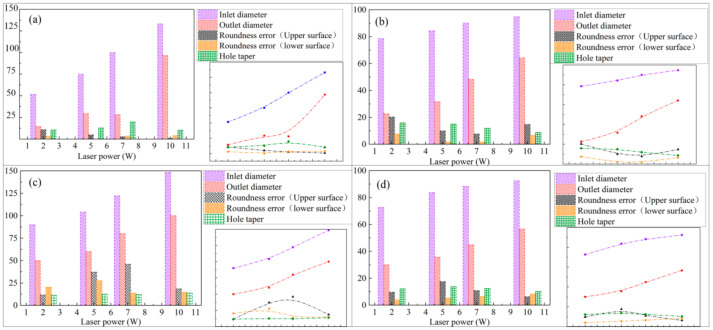
Hole appearance characteristics after fixed-position processing different metal materials: (**a**) aluminum alloy, (**b**) titanium alloy, (**c**) copper, (**d**) stainless steel.

**Figure 13:**
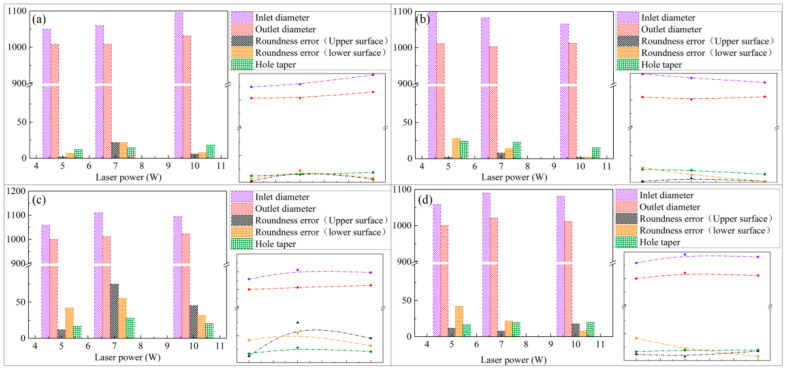
Hole appearance characteristics after helical drilling processing different metal materials: (**a**) aluminum alloy, (**b**) titanium alloy, (**c**) copper, (**d**) stainless steel.

**Figure 14:**
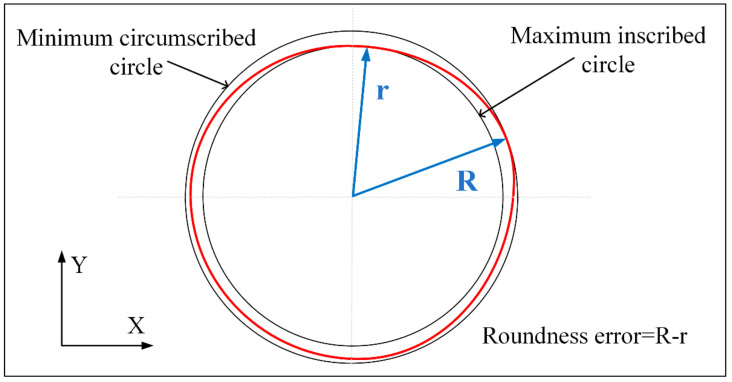
Roundness error calculation diagram.

In this paper, two kinds of different paths with 2 W, 5 W, 7 W, and 10 W laser power for four kinds of metal materials on drilling experiment, the samples are detected by Bruker Contour GT-K optical profiler. The test content includes the aperture of the entrance and exit surface, the roundness error of the entrance and exit surface, and the hole taper. The results are shown in Figure 12 and Figure 13. The minimum area method is used to evaluate the roundness error of the hole in this paper and that is, the radius difference of the two concentric circles containing the minimum circumscribed circle and the maximum inscribed circle of the measured circular profile is regarded as the roundness error, as shown in Figure 14.

From Figure 12 and Figure 13, it can be seen that when fixed-position drilling is used, the aperture of the upper and lower surfaces of the material has a significant upward trend with the increase of laser power, and the growth rate of the aperture of the upper and lower surfaces remains the same. The aperture of the helical drilling varies slightly with the increase of laser power, and the increase is not obvious. Due to water jet-guide laser drilling, the melting and vaporization of the processed material are the basic elements for forming the hole and the vaporization of the material mainly increases the depth of the hole. The melting of the hole wall material and the vapor pressure in the hole carry the molten material outward. The pulse frequency of the laser is fixed, the higher the laser power, the greater the laser pulse energy and the larger the aperture. During fixed-position drilling, the water jet has stagnant and backflow phenomena in the microporous. The cooling effect and chip removal ability of the material are not as good as the helical drilling. Heat accumulation is more serious, local rapid heating and the thermal deformation, which makes the hole roundness error more bigger. While the water jet can obtain a larger heat transfer area with the material in the helical drilling, keeping the aperture size better and reduce the roundness error. The roundness error of fixed-position drilling decreases with the increase of laser power and when the laser power is 10 W, the error of each material is reduced to within 10 µm.

In the experiment, the processing of copper materials showed special experimental results. Whether it is fixed-position processing or helical processing, the hole roundness error is always large, and the maximum error is more than 75 μm. This is because the thermal conductivity of copper is large, the laser reflectivity to this waveband is large, and the tension after melting is small. The fluidity is large, and the surface forming ability is poor [24]. Thus the impact of the water jet on its melting region affects the shape of the hole. The impact of the water jet is greater, and the chip removal efficiency of the water jet is also affected, making the microporous show an irregular shape.

**Figure 15:**
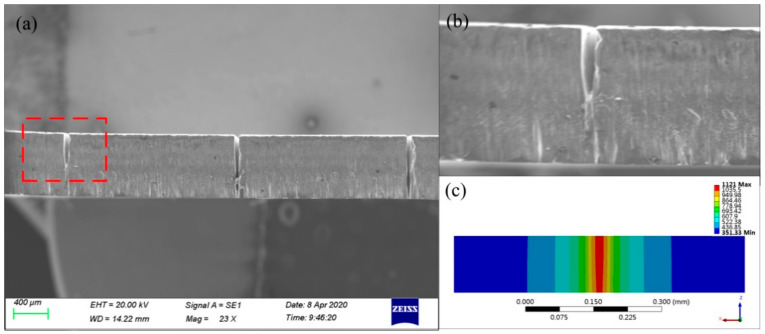
Comparison diagram of aluminum alloy hole shape between simulation results and experimental results of fixed-position drilling: (**a**) experimental result, (**b**) partial enlargement, (**c**) simulation result.

For processing schemes of different paths, the heat conduction of the laser energy for fixed-position drilling is always along the axial direction of the microporous, while the heat conduction of the spiral drilling laser energy is along the axial and radial directions of the microporous. The processing path makes the laser energy in the radial direction. It is dispersed during forward feeding, so the hole taper of fixed-position drilling is smaller than that of spiral drilling. With the increase of laser power, the taper of fixed-position drilling shows a decreasing trend, while the taper of helical drilling basically remains unchanged. Figure 15 shows the cross-sections micrographs of simulation and experiment of water jet-guided laser fixed-position drilling. It can be seen that there is no obvious heat-affected zone around the hole after processing and it has a large depth-to-diameter ratio and has a completely vertical hole wall shape within a certain depth. Compared with the use of helical processing, fixed-position drilling can obtain microporous with a smaller diameter. The processing size is completely determined by the diameter of the water jet, so it has important application value in processing smaller microporous. The simulation results are highly consistent with the experimental results in the prediction of the hole shape of each material, but there are deviations in the material removal rate and processing efficiency, because the model does not take into account the laser being absorbed by water and the water jet flow in the hole. The effect of material removal rate is larger than the experimental results, and the deviation reflects the complexity of the simulation.

## 5. Conclusions

- As the thermal effect is utilized in the WJGL machining, the temperature distribution is a crucial intermediate result that would determine the characteristics of final surfaces machined. Thus an important goal of the experimental studies carried out and presented in this work was to determine the unique heat conduction mechanism of the WJGL processing technology. This goal was accomplished by combining ANSYS finite element simulation with orthogonal experiments. In such a form, four typical metal materials were used for research. The influence of different laser power, scanning speed and processing paths on the hole shape and temperature distribution during processing is analyzed. The simulation results agree well with the experimental results. The obtained results of measurements and analyses allowed one to draw the following detailed conclusions: When water jet-guided laser processing technology is used to drill metal materials, the water jet can quickly cool the residual heat on the surface of the material and take away the processing residues, so that the surrounding area of the hole is clean and there is no heat-affected zone. The strong convective heat transfer effect of the water jet is significant, which plays an important role in ensuring the roundness of the hole. When determining the process parameters, in order to obtain a hole shape with a smaller roundness error, the use of small laser power should be avoided as much as possible.
- Copper, aluminum alloy, and other materials with low hardness and high thermal conductivity have more machining efficiency. For materials with high hardness and low thermal conductivity, such as titanium alloy and stainless steel, the hole roundness is better, the hole edge temperature is lower, and the thermal deformation is smaller. From this, it can be inferred that water jet-guided laser processing materials with high hardness and low thermal conductivity have good drilling ability, ensuring a good hole roundness, which is conducive to forming a hole structure with a flat bottom and a vertical side wall, and has high precision processing ability.
- Hole roundness error and hole taper decrease with the increase of laser power. The roundness error of each material is reduced to within 10 μm when the laser power is 10 W, and the average hole taper is 8.73°. Compared with helical drilling, fixed-position drilling can obtain a smaller hole taper, and have high processing efficiency. It does not change the relative position of the workpiece and the laser source during processing, and has important application value in processing small microporous.

## Figures and Tables

**Table 1:** The chemical composition of materials (mass fraction, %).

**6061 aluminum alloy**	Cu	Si	Fe	Mn	Mg	Zn	Cr	Ti	Al
	0.15~0.40	0.40~0.80	0.70	0.15	0.80~1.20	0.25	0.04~0.35	0.15	the rest
**TC4 titanium alloy**	Fe	C	N	H	O	Al	V	Ti	
	≤0.30	≤0.10	≤0.05	≤0.015	≤0.20	5.50~6.80	3.50~4.50	the rest	
**Pure copper**	Cu + Ag	Bi	Pb	Fe	Sb	S	As		
	≥99.90	≤0.001	≤0.005	≤0.005	≤0.002	≤0.005	≤0.002		
**304 stainless steel**	C	Si	Mn	Ni	Cr	S	P	Fe	
	≤0.08	≤1.00	≤2.00	8.00~11.00	18.00~20.00	≤0.03	≤0.045	the rest	

**Table 2:** Thermal performance parameters of materials.

Item	Aluminum Alloy (6061)	Titanium Alloy (TC4)	Copper (Cu)	Stainless Steel (304)
Density (kg/m^3^)	2713	4620	8300	7850
Thermal conductivity (W/(m·K))	155.3	21.9	401	60.5
Specific heat constant (J/(kg·K))	915.7	523	385	434
Melting point (K)	823	1951	1357	1713
Boiling point (K)	2327	4623	2835	4912
Reflection coefficient	0.91	0.63	0.99	0.64

**Table 3:** Simulation parameters of personal computer (PC).

Setting Parameters	Fixed-Position Drilling	Spiral Drilling
Average laser power (W)	2, 5, 7, 10	2, 5, 7, 10
Pulse frequency (kHz)	40, 80	40, 80
Pulse duration (μs)	0.3	0.3
Pulse period (s)	0.00001	0.00001
Drilling time (s)	1, 2, 4, 6, 8, 10	/
Spiral cutting speed (mm/s)	/	0.8, 1.0, 1.2
Max outer circle (mm)	/	1
Number of mesh	143,352	619,544
Convection heat transfer coefficient [W/(mm^2^·K)]	0.14 (top surface)	0.14 (top surface)
	0.08 (other surfaces except the top)	0.08 (other surfaces except the top)

**Table 4:** The relative parameters of water jet heat transfer coefficient are calculated [23].

Parameter	Symbol	Value
Thermal conductivity of water	λ	0.6 (W/m °C)
The moving viscosity coefficient of water	υ	1.01 × 10^−6^ (m^2^/s)
Kinetic viscosity coefficient of water	η	1.01 × 10^−3^ (Pa/s)
The heat capacity of water	$c_{w}$	4.2 × 10^3^ (J/kg·℃)
The density of water	$\rho_{w}$	1000 (kg/m^3^)
Water pressure	$P_{w}$	20 (MPa)
Pressure loss coefficient	φ	0.995
Water velocity	V	199 (m/s)
Prandtl number	Pr	7.07

## Nomenclature

WJGL	Water jet-guide laser processing technology
EDM	Electrical Discharge Machining
Nd-YAG	Neodymium-doped Yttrium Aluminum Garnet
PC	Personal computer
CCD	Charge coupled device
ρ	Density, kg/m^3^
C	Specific heat, J/(kg·K)
λ	Thermal conductivity, W/(m·K)
n	Normal vector of surface
$h_{1}$	Water severe convection heat transfer coefficient, W/(mm^2^·K)
$h_{2}$	Water natural convection heat transfer coefficient, W/(mm^2^·K)
T	Cell temperature
$T_{\infty }$	Ambient temperature
q	Laser absorption heat flux, W/mm^2^
P	Peak power of pulsed laser, W
D	Diameter of water jet, m
t	Processing time, s
Re	Reynold number
Pr	Prandtl number
r	Distance between heat transfer zone and stagnation point, m
V	Flow velocity, m/s
υ	Motion viscosity coefficient of water, m^2^/s
η	Kinetic viscosity coefficient of water, Pa·s
$c_{w}$	Specific heat capacity of water, J/kg·℃
$\rho_{w}$	Density of water, kg/m^3^
$P_{w}$	Water pressure, MPa
φ	Pressure loss factor
Nμ	Nusselt number
h	Convection heat transfer coefficient, W/(mm^2^·K)
z	Depth of laser action, m
$F_{0}$	Peak power density (W/m^2^)
R	Reflectivity of the material surface
A	Spot area, m^2^

## References

[1] Zhu S.J., Zhang C.Y., Chu S.L., Yang Z.Y., Zhang X.S., Wang A.B. (2020). Research and application of mass microporous water-assisted picosecond laser processing technology. Chin. J. Lasers.

[2] Chichkov B.N., Momma C., Nolte S., Von Alvensleben F., Nnermann A. (1996). Femtosecond, picosecond and nanosecond laser ablation of solids. Appl. Phys. A Mater. Sci. Process..

[3] Xie B.C., Cui H.X., Zhang Y., Liu X.L. (2018). Current research of micro electrical discharge machining of micro-hole. J. Harbin Univ. Sci. Technol..

[4] Zhu Y.D. (2018). Analysis of small diameter deep hole machining and micropore machining characteristics in electrical discharge machining. China Met. Form. Equip. Manuf. Technol..

[5] Wang Y.Y., Jia C., Xu M., Geng X.P. (2018). The prospect of micro hole processing technology. Hydraul. Pneum. Seals.

[6] Jia J.X. (2011). Research on the Machining of Micro Holes by Electrohydraulic Drilling based on Sodium Nitrate. Ph.D. Thesis.

[7] Jiao Y., He B., Li P., Tian D.P. (2018). Development of micro holes machining with high aspect ratio. Aeronaut. Sci. Technol..

[8] Xia B., Jiang L., Wang S.M., Yan X.L., Liu P.J. (2013). Femtosecond laser drilling of micro holes. Chin. J. Lasers.

[9] Perry M., Stuart B., Banks P., Feit M., Rubenchik A. (1999). Ultrashort-pulse laser machining of dielectric materials. J. Appl. Phys..

[10] Zhang Y.N., Qiao H.C., Zhao J.B., Cao Z.H., Yu Y.F. (2020). Numerical simulation of water jet–guided laser micromachining of CFRP. Mater. Today Commun..

[11] Kalyanasundaram D., Shehata G., Neumann C., Shrotriya P., Molian P. (2008). Design and validation of a hybrid laser/water-jet machining system for brittle materials. J. Laser Appl..

[12] Sutowska M., Pimenov D.Y., Gupta M.K., Mia M., Sharma S. (2020). Influence of variable radius of cutting head trajectory on quality of cutting kerf in the abrasive water jet process for soda–lime glass. Materials.

[13] Yang C.M., Jiang T., Yu Y.Q., Dun G., Ma Y., Liu J. (2018). Study on surface quality of wood processed by water-jet assisted nanosecond laser. Bioresources.

[14] Barcikowski S., Koch G., Odermatt J. (2006). Characterisation and modification of the heat affected zone during laser material processing of wood and wood composites. Holzals Roh-Und Werkst..

[15] Chen X., Su Y.C., Wang Y.Q., Yang D., Yang Y.P., Feng S. (2009). Study on nonlinear transmission characteristics of ultrashort laser pulses in water. J. Opt..

[16] Lu X.Z., Jiang K.Y., Jiang F., Lei T.P. (2015). Micro-jet flow conduction improves the energy distribution of machining laser. Appl. Laser.

[17] Sun D., Wang J.H., Han F.Z. (2016). Contrastive study of water jet-guided laser and water jet assisted laser cutting of monocrystalline silicon. Appl. Laser.

[18] Zhao W.N., Huang Y.H., Song H.W., Huang C.G. (2017). Multi-Scale Analysis Model of Thermal-Mechanical Damage Effect in High-Power Continuous-Wave Laser Irradiation of CFRP Laminates. Chin. J. Lasers.

[19] Sun D., Han F., Ying W. (2019). Numerical simulation of water jet–guided laser cutting of carbon fiber–reinforced plastics. Proc. Inst. Mech. Eng. Part B J. Eng. Manuf..

[20] Couty P., Wagner F., Hoffmann P. (2005). Laser coupling with a multimode water-jet waveguide. Opt. Eng..

[21] Liu X., Lienhard J.H., Lombara J.S. (1991). Convective heat transfer by impingement of circular liquid jets. J. Heat Transf..

[22] Gabour L.A. (1991). Heat Transfer to Turbulent and Splattering Impinging Liquid Jets.

[23] Guan Z.Z. (2007). Laser Machining Process Manual.

[24] Zhao Y., Feng A.X., Yang H.H., Chen H. (2018). Effect of laser texture on infrared laser absorption of copper surface. Surf. Technol..

